# Are ICD-10 codes appropriate for performance assessment in asthma and COPD in general practice? Results of a cross sectional observational study

**DOI:** 10.1186/1472-6963-5-11

**Published:** 2005-02-01

**Authors:** Antonius Schneider, Lutz Gantner, Inko Maag, Mathias M Borst, Michel Wensing, Joachim Szecsenyi

**Affiliations:** 1 Department of General Practice and Health Services Research, Bergheimer Strasse 147, D-69115 Heidelberg, Germany; 2 Caritas Medical Hospital I, Uhlandstrasse 7, D-97980 Bad Mergentheim, Germany

## Abstract

**Background:**

The increasing prevalence and impact of obstructive lung diseases and new insights, reflected in clinical guidelines, have led to concerns about the diagnosis and therapy of asthma and COPD in primary care. In Germany diagnoses written in medical records are used for reimbursement, which may influence physicians' documentation behaviour. For that reason it is unclear to what respect ICD-10 codes reflect the real problems of the patients in general practice. The aim of this study was to assess the appropriateness of the recorded diagnoses and to determine what diagnostic information is used to guide medical treatment.

**Methods:**

All patients with lower airway symptoms (n = 857) who had attended six general practices between January and June 2003 were included into this cross sectional observational study. Patients were selected from the computerised medical record systems, focusing on ICD-10-codes concerning lower airway diseases (J20-J22, J40-J47, J98 and R05). The performed diagnostic procedures and actual medication for each identified patient were extracted manually. Then we examined the associations between recorded diagnoses, diagnostic procedures and prescribed treatment for asthma and COPD in general practice.

**Results:**

Spirometry was used in 30% of the patients with a recorded diagnosis of asthma and in 58% of the patients with a recorded diagnosis of COPD. Logistic regression analysis showed an improved use of spirometry when inhaled corticosteroids were prescribed for asthma (OR = 5.2; CI 2.9–9.2) or COPD (OR = 4.7; CI 2.0–10.6). Spirometry was also used more often when sympathomimetics were prescribed (asthma: OR = 2.3; CI 1.2–4.2; COPD: OR = 4.1; CI 1.8–9.4).

**Conclusions:**

This study revealed that spirometry was used more often when corticosteroids or sympathomimetics were prescribed. The findings suggest that treatment was based on diagnostic test results rather than on recorded diagnoses. The documented ICD-10 codes may not always reflect the real status of the patients. Thus medical care for asthma and COPD in general practice may be better than initially found on the basis of recorded diagnoses, although further improvement of practice patterns in asthma and COPD is still necessary.

## Background

There is broad agreement between existing guidelines for asthma [1-4] regarding diagnostic procedures, patient education and medical treatment [5]. The statements for chronic obstructive pulmonary disease (COPD) [6-8] are also consistent, despite the lack of evidence for some recommendations [9]. However, it has been shown previously that the management of these diseases in practice was not fully consistent with the guidelines [10-12], which may be related to the variety of views on the treatment of these conditions [13,14]. Furthermore, the knowledge about the diseases is changing rapidly and the management has become more complex [15,16]. Many patients with asthma or COPD are managed in primary care and it is important to optimise the treatment given the prevalence and impact [17].

However, it remains difficult to draw a clear picture of management of obstructive lung diseases in primary care since the diagnoses of asthma and COPD often have not been separated in many surveys [7]. This could be due to some diagnostic and therapeutic overlap between these both diseases. Especially in primary care it is difficult to distinguish both entities when the symptoms are presented in an early stage [18]. In particular the treatment response in this stage often seem to be not strongly related to the stated diagnoses [19,20], and also the treatment response of COPD in the long term course is disappointing [21].

Furthermore, systematic registration of diseases in primary care are often hampered by missing of recorded diagnoses [22] or discrepancies between diagnoses and prescribed medications [23]. A specific problem for health services research and performance assessment in Germany might be that diagnoses saved in medical records are used for reimbursement. For that reason it is unclear to what respect ICD-10 codes reflect the real problems of patients in general practice. It may be possible that physicians enter diagnoses in their records to get reimbursement, but use other information such as results of diagnostic tests for treatment. Empirical evidence on this phenomenon is limited, but its existence would complicate the assessment of practice patterns.

The aim of this study was to assess the appropriateness of the recorded diagnoses and to determine what diagnostic information is used to guide medical treatment. For this purpose we analysed associations between recorded diagnoses, diagnostic procedures and medical treatment regarding asthma and COPD in general practice.

## Methods

### Study design

A cross-sectional observational study was performed in six computerised general practices with a total of eleven GPs in the Rhine-Neckar region in Southern Germany.

### Setting and study subjects

All patients who had attended their physicians with asthma, COPD or other lower airway diseases from January until June 2003 were included in the study.

### Ethical approval

The study was approved by the Medical Ethics Committee of the University of Heidelberg.

### Procedure for selection of subjects

Patients were identified by searching the electronic files for documented diagnoses concerning lower airway diseases within the time frame. The data were evaluated systematically according to the international classification of diseases (ICD-10). In detail, the practice software was checked for the ICD-10-codes of the chapter "chronic lower airway disease": J40 (bronchitis, not specified as acute or chronic), J41 (simple and mucopurulent chronic bronchitis), J42 (unspecified chronic bronchitis), J43 (emphysema), J44 (chronic obstructive pulmonary disease), J45 (asthma), J46 (status asthmaticus), J47 (bronchiectasis). Additionally, the ICD-10-codes J98 (diseases of bronchus, not elsewhere classified) and R05 (cough / bronchial hyperreactivity), and the repeated documentation of ICD-10-codes belonging to the chapter "acute lower airway disease" were extracted in order to detect potentially existing but not detected asthma or COPD. The chapter "acute lower airway disease" includes the diagnoses J20 (acute bronchitis), J21 (acute bronchiolitis) and J22 (unspecified acute lower respiratory infection).

The performed diagnostic procedures and the actual medication for each identified patient were extracted and documented manually by two independent scientific fellows (L.G., I.M.) of the department. The variables of the used protocol for documentation are listed in table 2.

**Table 1:** Baseline characteristics

	**Overall (n = 857)**	**Asthma (n = 255)**	**COPD (n = 112)**	**Asthma + COPD (n = 25)**	**Other (n = 465)**
age	41.84 ± 22.18 (min 1; max 97)	36.48 + 21.00 (min 1; max 88)	56.28 + 18.38 (min 21; max 92)	46.23 + 19.16 (min 26; max 71)	41.06 + 22.32 (min 1; max 97)
sex	m 385; f 472	m 121; f 134	m 55; f 57	m 15; f 10	m 194; f 271

### Analysis

Every ICD10-code specified above was extracted and written down, at maximum up to three ICD-10-Codes per patient were found. The diagnoses were clustered into three groups: asthma, COPD, and other lower airway diseases. If more than one diagnosis was documented, asthma (J45) or COPD (J44) were extracted as the leading diagnosis.

The ICD-10 group J43 (emphysema) was included into the ICD-10 group J 44 (COPD). The ICD-10 groups other than J45 (asthma), J44 or J43 were merged into the group "other lower airway diseases".

The performed diagnostic procedures were extracted manually. Four ways of making diagnoses have been found in the records: 1. taking medical history only, 2. taking medical history plus trial of medication, 3. taking medical history and performing spirometry, 4. taking medical history plus single measurement of PEF. The additional performance of bronchial challenge testing and chest X-ray was documented, too.

Baseline data were evaluated descriptively. The t-test was used to detect differences of age. Linear logistic regression was used to test relationships between diagnosing asthma or COPD and the performed diagnostics. The relation between prescribing patterns and performed diagnostics was also examined with logistic regression analysis.

## Results

Overall, 8765 patients attended the practices within the time frame. 857 (9,8%) patients had lower airways diseases (385 male, 472 female).

A total of 255 (2.9% of 8765) had the recorded diagnosis of asthma and 112 (1.3% of 8765) had COPD (107 with J44 = COPD, 5 with J43 = emphysema). A combination of asthma and COPD was documented in 25 (0.2% of 8765) cases.

The 465 (5.3% of 8765) patients with "other lower airway diseases" included seven cases with acute bronchitis (J20), none with acute bronchiolitis (J21), one case with unspecified acute lower respiratory infection (J22), 251 (2.9% of 8765) cases with not specified bronchitis (J40), 38 with simple and mucopurulent chronic bronchitis (J41), 57 with unspecific chronic bronchitis (J42), one with bronchiectasis (J47), seven with diseases of bronchus, not classified (J98), 103 (1.2% of 8765) with cough / bronchial hyperreactivity (R05), Patients with COPD were significantly older than those with asthma or other lower airway diseases. There was no case of twofold documentation of "acute lower airway disease" (J20 - J22) within the time frame. The details regarding the patient population are described in table 1.

**Table 2:** Performed diagnostics and documented diagnoses

	**Asthma (n = 255)**				**COPD (n = 112)**				**Other (n = 465)**			
**Performed diagnostics**	**n**	**%**	**OR**	**95% CI**	**n**	**%**	**OR**	**95% CI**	**n**	**%**	**OR**	**95% CI**
Medical history only	42	16.5	**0.11**	0.07–0.15	33	29.3	**0.31**	0.20–0.46	338	72.7	**10.9**	7.89–15.02
Medical history and trial of medication	57	22.4	**8.11**	4.68–14.08	6	5.4	0.51	0.23–1.13	12	2.6	**0.14**	0.07–0.26
Medical history and spirometry	77	30.2	1.30	0.99–1.72	65	58.1	**4.38**	2.99–6.40	90	19.4	**0.37**	0.27–0.50
Medical history and PEF-measurement	79	31.0	**7.57**	4.93–11.60	8	7.1	0.81	0.47–1.40	25	5.4	**0.18**	0.11–0.28
Additional bronchial challenge test	18	3.9	1.35	0.73–2.48	24	21.4	**6.74**	3.66–12.42	1	0	**0.16**	0.08–0.35
Additional chest X-ray	34	13.3	0.88	0.58–1.31	42	37.5	**4.06**	2.67–6.19	48	10.3	**0.44**	0.30–0.65

### Diagnostics

Table 2 shows the performed diagnostic procedures. Spirometry was performed in 30% of the patients with recorded asthma. A high amount was diagnosed with 'medical history and single measurement of peak expiratory flow' (31%), and 22% of cases fell into the category of medical history taking plus trial of medication (= diagnosing by therapy). This implies that the physician gives sympathomimetics in suspected asthma. If the patient feels better, the diagnosis 'asthma' is made.

In about 17% the diagnosis asthma was made only on the basis of medical history

Spirometry was performed in 58% of the cases with recorded COPD. In 29% the diagnosis was made only by medical history. A small percentage (7%) was diagnosed by history taking and PEF-measurement and 5% by diagnosing by therapy.

Concerning the other lower airway diseases, spirometry was done in 19% in order to exclude asthma or COPD. These diagnoses were made mainly on the basis of medical history (73%).

The associations between recorded diagnoses and diagnostic procedures are calculated by binary logistic regression (also in table 2). The higher the odds ratio (OR) the higher is the positive association between the variables. Patients with the combined diagnosis of asthma and COPD were not included into the analysis. An odds ratio smaller than one means that there is a negative association between those variables. Significant results are bold.

The logistic regression showed that the diagnosis asthma was often based only on single measurement of PEF (OR 7.6) or 'trial of medication' (OR 8.1). The use of spirometry was not significantly associated with the recorded diagnosis asthma.

Concerning the recorded diagnosis of COPD, 'trial of medication' was not associated significantly. Instead, the recorded diagnosis was based on spirometry (OR 4.4). Bronchial challenge testing was used to exclude asthma (OR 6.7). Also chest X-ray showed a significant association with the recorded diagnosis of COPD (OR 4.1).

The recording of other lower airway diseases was mainly based on the medical history (OR 10.9), the other diagnostic procedures are used only in few cases (OR < 1).

### Prescriptions

The documentation of the prescribed medication showed that 66% of patients with asthma and 42% with COPD received sympathomimetics (table 3). 38% of the asthma patients received inhaled corticosteroids, whereas 46% of the patients with COPD were treated with inhaled corticosteroids. Cromoglycate was given in 36% of patients with asthma and 14% of COPD.

**Table 3:** Relationship between performed diagnostis. actual treatment and documented diagnoses

**Performed diagnostics and actual treatment**	**Asthma (n = 255)**				**COPD (n = 112)**				**Other (n = 465)**			
	**n**	**%**	**OR**	**95% CI**	**n**	**%**	**OR**	**95% CI**	**n**	**%**	**OR**	**95% CI**

**Sympathomimetics**	**(n = 167; 65.5%)**				**(n = 47; 42.0%)**				**(n = 36; 7.7%)**			
Medical history only	9	3.5	**0.22**	0.10–0.51	7	6.3	1.35	0.56–3.26	10	2.2	**4.97**	2.05–12.02
Medical history and trial of medication	52	20.4	**7.99**	3.06–20.85	4	3.6	2.93	0.51–16.71	8	1.7	**31.48**	8.95–111.2
Medical history and spirometry	60	23.5	**2.28**	1.24–4.18	36	32.1	**4.06**	1.77–9.35	14	3.0	**3.10**	1.51–6.36
Medical history and PEF-measurement	46	18.0	0.67	0.43–1.07	0	0	0	0-0	4	1.0	2.39	0.77–7.36
Additional bronchial challenge test	12	4.7	3.51	0.77–16.04	9	8.0	1.16	0.44–3.08	0	0	0.01	0–10^9^
Additional chest X-ray	22	8.6	1.02	0.48–2.17	22	19.6	1.98	0.91–4.31	6	1.0	1.91	0.75–4.86
												
**Corticosteroids**	**(n = 96; 37.6%)**				**(n = 52; 46.4%)**				**(n = 28; 6.0%)**			
Medical history only	4	1.6	**0.23**	0.08–0.66	9	8.0	2.22	0.85–5.78	5	1.0	2.37	0.78–7.23
Medical history and trial of medication	20	7.8	0.91	0.49–1.68	3	2.7	1.16	0.22–6.03	5	1.05	**13.99**	4.11–47.64
Medical history and spirometry	49	19.2	**5.17**	2.91–9.19	40	35.7	**4.67**	2.05–10.64	15	3.2	**6.05**	2.72–13.45
Medical history and PEF-measurement	23	9.0	0.58	0.33–1.02	0	0	0	0–14 × 10^4^	3	1.0	2.26	0.63–8.04
Additional bronchial challenge test	9	3.5	**3.30**	1.07–10.17	16	14.3	**6.22**	1.93–20.11	0	0	0.00	0-0
Additional chest X-ray	16	6.3	1.63	0.79–3.37	29	25.9	**4.56**	2.00–10.38	10	2.2	**6.19**	2.65–14.47
												
**Cromoglycate**	**(n = 91; 35.7%)**				**(n = 16; 14.3%)**				**(n = 39; 8.4%)**			
Medical history only	9	3.5	**0.16**	0.07–0.37	1	1.0	0.23	0.05–1.02	21	4.5	**12.02**	4.93–29.29
Medical history and trial of medication	24	9.4	1.52	0.83–2.79	0	0	0.00	0–1.6 × 10^18^	4	1.0	**6.16**	1.77–21.51
Medical history and spirometry	23	9.0	0.74	0.42–1.32	10	8.9	1.07	0.40–2.86	10	2.2	1.70	0.89–3.25
Medical history and PEF-measurement	35	13.7	**1.74**	1.01–3.00	5	4.5	**14.1**	2.96–67.18	4	1.0	2.17	0.71–6.65
Additional bronchial challenge test	5	2.0	1.06	0.34–3.26	4	3.6	1.67	0.48–5.83	1	0	1.62	0.19–13.54
Additional chest X-ray	9	3.5	0.65	0.29–1.46	5	4.5	0.73	0.23–2.25	8	1.7	**2.58**	1.11–6.01

Only a small amount of patients with 'other lower airway diseases' received anti-obstructive medicine (6–8%).

The logistic regression showed that sympathomimetics were given in asthma when having shown efficacy (trial of medication) (OR 8.0) or when spirometry was performed (OR 2.3). Therapy with corticosteroids was significantly associated with spirometry (OR 5.2) and bronchial challenge testing (OR 3.3).

Treatment of COPD with sympathomimetics was significantly associated with the use of spirometry (OR 4.1). Inhaled corticosteroids were also given more often when spirometry (OR 4.7) and Chest X-ray (OR 4.6) had been performed. Bronchial challenge testing was used to exclude asthma (OR 6.2).

In asthma as well as in COPD corticosteroids are not prescribed only by trial of medication and are not given after making the diagnosis only by medical history.

Prescribing of cromoglycate was associated with PEF measurement in both asthma and COPD (OR 1.7 respectively 14.1)

Treatment of the other lower airway diseases with sympathomimetics respectively corticosteroids was mainly associated with testing the efficacy of therapy (OR 31.5 respectively 14.0). The prescription is lower associated with spirometry (OR 3.1 respectively 6.1) or x-ray (OR 1.9 respectively 6.2). Cromoglycates were given mostly on the basis of 'taking medical history only' (OR 12.0) and trial of medication (OR 6.2).

## Discussion

Although guidelines recommend the use of spirometry, this was used in only in 30% of the patients with a recorded diagnosis of asthma and in 58% of the patients with a recorded diagnosis of COPD. A high number of recorded diagnoses of asthma and COPD were insufficiently diagnosed by PEF-measurement, medical history and diagnosing by therapy. On the first sight, patients with asthma seem to be treated insufficiently with anti-inflammatory therapy and patients with COPD seemed to be over-treated with steroids.

However, further analysis showed a high association between prescribed corticosteroids and sympathomimetics as long term medication and performed diagnostic procedures. This improvement of diagnostic investigation when potential harmful drugs are given demonstrates that treatment of asthma and COPD by the GPs may be more consistent with guidelines than other studies have found [10,13]. This hypothesis is facilitated by the fact, that cromoglycates seem to be administered if there was diagnostic uncertainty, as there was no significant association between performing spirometry and making diagnoses in those cases.

According to this, our study shows that a deeper look insight with analysis of diagnoses together with performed diagnostics and medication could prove a better care for people with obstructive airway diseases than a single analysis of the documented diagnoses. As a conclusion, ICD-10 codes seem not to be able to reflect the work in primary care, in particular if the difficulties of the diagnostic and therapeutic differentiation between asthma and COPD in earlier stages are taken into account [18,20].

Therefore it seems that the documented diagnosis due to ICD-10 often serves as a 'working hypothesis'. The needs of family physicians for practice and research would be best met by the International Classification of Primary Care (ICPC-2) [24], but the general practitioners in Germany are politically obliged to record ICD-10 diagnoses to get reimbursement. As the documentation of the reason of encounter is restricted in these terms of ICD-codes there could be some necessity to assign diagnoses although they do not match with the real status of the patient. This makes the recorded diagnoses unreliable, sometimes resulting in apparently poor coherence between medication and diagnoses, and this leads to difficulties in drawing valid conclusions on the quality of primary care.

Another critical point is, that the 'trial of medication' as a 'fact of life in primary care' could implicate a patients' perception of improvement which could lead the doctor to perpetuate the therapy, thus resulting in over-prescribing accompanied by an avoidable risk of side-effects. Especially this 'trial of medication' may reflect diagnostic uncertainty in general practice, which must normally have consequences for the classification and labelling. In absence of a reasonable classification system the GPs are forced to hide this uncertainty by documenting diagnoses in ICD-10-codes. As a result, GPs could feel the uncertainty nevertheless, but start seeing it as a personal shortcoming rather than a reality of medicine. Therefore this exaggeration of 'labelling with ICD-10' could also constrain a systematic quality improvement in diagnostic performance in primary care.

The strength of the study was the detailed analysis with manual extraction and documentation of performed diagnostics and actual medication of each identified patient. As the ICD-10 classification made it difficult to identify patients with obstructive airway diseases in junction with performed diagnostics and therapy we searched all records manually. Because of this complexity the study had the limitation that the number of GPs was small, which reduces the generalisability of the descriptive findings, although the estimates of associations are less sensitive for this problem.

The prevalence of asthma and COPD in this German sample of general practice seems to be low compared to the prevalence of about 5% in studies of other countries [25-27]. One explanation might be the high doctor-patient contact rate in Germany that decreases the prevalence of severe diseases in general practice.

## Conclusions

The conclusion of this study is that performance assessment should take the reliability problems of recorded diagnoses into account, especially if the ICD-10 classification is used. A more detailed analysis may be necessary for adequate evaluation of primary care, consequential pointing out to the fact, that treatment of obstructive airways diseases by the GP's may be more consistent with guidelines than other studies have found. Despite these findings improvement of diagnostic performance in general practice is necessary, because a routine use of spirometry is of growing importance against the background of the increasing prevalence of obstructive airway diseases.

## Competing interests

The author(s) declare that they have no competing interests.

## Authors contributions

AS conceived and designed the study, wrote the report. LG and IM checked the practice software and documented data. MB helped with interpreting the data and writing the report. MW helped with interpreting the data and writing the report. JS helped with revising the report. All authors read and approved the final manuscript.

## Pre-publication history

The pre-publication history for this paper can be accessed here:


